# Supplementary material for: DNA Polymorphisms and mRNA Levels of Immune Biomarkers as Candidates for Inflammatory Postpartum Disorders Susceptibility in Italian Buffaloes
Source: Vet Sci. 2023 Sep 14;10(9):573. doi: 10.3390/vetsci10090573 (PMC10534879; doi:10.3390/vetsci10090573)
Supplement: Supplementary file 1 [file vetsci-10-00573-s001.zip › vetsci-2598179-supplementary.pdf]

Supplementary:

```
XM_044936650.2      CCGGCTTGGACAGCCTCTCTTGTACCTTCCGCCGGCCGGCCAGGCAGGGGCGGGACCTGA      60
H                   CCGGCTTGGACAGCCTCTCTTGTACCTTCCGCCGGCCGGCCAGGCAGGGGCGGGACCTGA      60
RD                  CCGGCTTGGACAGCCTCTCTTGTACCTTCCGCCGGCCGGCCAGGCAGGGGCGGGACCTGA      60
                    ************************************************************

XM_044936650.2      GCGTTTTACTTCCGGATCCCACAGCTTAGACACCGGAAGCCGGATGTGAGAAGTTGGCAG      120
H                   GCGTTTTACTTCCGGATCCCACAGCTTAGACACCGGAAGCCGGATGTGAGAAGTTGGCAG      120
RD                  GCGTTTTACTTCCGGATGCCACAGCTTAGACACCGGAAGCCGGATGTGAGAAGTTGGCAG      120
                    ***************** ******************************************

XM_044936650.2      CGGGAGAAGGGAAGGGCGGCAGCGGGAGAAGGGAAGGGCTACAGCGGAACTCGGACTTTC      180
H                   CGGGAGAAGGGAAGGGCGGCAGCGGGAGAAGGGAAGGGCTACAGCGGAACTCGGACTTTC      180
RD                  CGGGAGAAGGGAAGGGCGGCAGCGGGAGAAGGGAAGGGCTACAGCGGAACTCGGACTTTC      180
                    ************************************************************

XM_044936650.2      TCGGAGCGCCGGGGGTCCGAGGAACGCTCGGGGCCCGCCGTTCCCGCCCTTCTCACGTCG      240
H                   TCGGAGCGCCGGGGGTCCGAGGAACGCTCGGGGCCCGCCGTTCCCGCCCTTCTCACGTCG      240
RD                  TCGGAGCGCCGGGGGTCCGAGGAACGCTCGGGGCCCGCCGTTCCCGCCCTTCTCACGTCG      240
                    ************************************************************

XM_044936650.2      GACCGACTCAGCTGACAGGCCCTGACGTGTTGGATGAGCAGGCCCCCCTGGAAGAGTCCA      300
H                   GACCGACTCAGCTGACAGGCCCTGACGTGTTGGATGAGCAGGCCCCCCTGGAAGAGTCCA      300
RD                  GACCGACTCAGCTGACAGGCCCTGACGTGTTGGATGAGCAGGCCCCCCTGGAAGAGTCCA      300
                    ************************************************************

XM_044936650.2      CTGTGTGAGATGGTGCAGCCGAGTGGCGGTCCGGCAGGGGACCAGGACATGCTGGGTGAA      360
H                   CTGTGTGAGATGGTGCAGCCGAGTGGCAGTCCGGCAGGGGACCAGGACATGCTGGGTGAA      360
RD                  CTGTGTGAGATGGTGCAGCCGAGTGGCGGTCCGGCAGGGGACCAGGACATGCTGGGTGAA      360
                    *************************** ********************************

XM_044936650.2      GAGTCTTCTCTGGGGAAGCCAGCCATGCTCCATGTGCCTTCAGAGCAGGGCA    412
H                   GAGTCTTCTCTGGGGAAGCCAGCCATGCTCCATGTGCCTTCAGAGCAGGGCA    412
RD                  GAGTCTTCTCTGGGGAAGCCAGCCATGCTCCATGTGCCTTCAGAGCAGGGCA    412
                    ****************************************************
```

Figure S1. Analysis of nitrogenous bases matching for DNA in healthy and reproductive disorders affected buffaloes using GenBank gb|XM_044936650.2| and *IKBKG* marker (412-bp) sequences.

```
XM_025282898.3      CTCATTTCTCCTGCAGAGTCTTCAGACAGCTGGTGCCGAAGTTTCAGGAACATCCTCCTC      60
H                   CTCATTTCTCCTGCAGAGTCTTCAGACAGCTGGTGCCGAAGTTTCAGGAACATCCTCCTC      60
RD                  CTCATTTCTCCTGCAGAGTCTTCAGACAGCTGGTGCCGAAGTTTCAGGAACATCCTCCTC      60
                    ************************************************************

XM_025282898.3      TCTTCAATCATGGCTTGTGGTCTGGTCGCCAGCAACCTGAATCTCAAACCTGGGGAGTGC      120
H                   TCTTCAATCATGGCTTGTGGTCTGGTCGCCAGCAACCTGAATCTCAAACCTGGGGAGTGC      120
RD                  TCTTCAATCATGGCTTGTGGTCTGGTCGCCAGCAACCTGAATCTCAAACCTGGGGAGTGC      120
                    ************************************************************

XM_025282898.3      CTCAGAGTGCGGGGCGAGGTGGCCGCAGACGCCAAGAGCTTCTTGCTGAACCTGGGCAAA      180
H                   CTCAGAGTGCGGGGCGAGGTGGCCGCAGACGCCAAGAGCTTCTTGCTGAACCTGGGCAAA      180
RD                  CTCAGAGTGCGGGGCGAGGTGGCCGCAGACGCCAAGAGCTTCTTGCTGAACCTGGGCAAA      180
                    ************************************************************

XM_025282898.3      GATGACAACAATCTGTGCCTCCACTTCAACCCTCGTTTCAACGCGCATGGGGACATCAAC      240
H                   GATGACAACAACCTGTGCCTCCACTTCAACCCTCGTTTCAACGCGCATGGGGACATCAAC      240
RD                  GATGACAACAATCTGTGCCTCCACTTCAACCCTCGTTTCAACGCGCATGGGGACATCAAC      240
                    *********** ************************************************

XM_025282898.3      ACCATTGTGTGTAACAGCAAGGACGCTGGGGCCTGGGGGACCGAGCAGAGGGAATCTGCC      300
H                   ACCATTGTGTGTAACAGCAAGGACGCTGGGGCCTGGGGGACCGAGCAGAGGGAATCTGCC      300
RD                  ACCATTGTGTGTAACAGCAAGGACGCTGGGGCCTGGGGGACCGAGCAGAGGGAATCTGCC      300
                    ************************************************************

XM_025282898.3      TTCCCCTTCCAGCCTGGAAGTGTCGTGGAGGTATGCATCTCCTTCGACCAGACAGACCTA      360
H                   TTCCCCTTCCAGCCTGGAAGTGTCGTGGAGGTATGCATCTCCTTCGACCAGACAGACCTA      360
RD                  TTCCCCTTCCAGCCTGGAAGTGTCGTGGAGGTATGCATCTCCTTCGACCAGACAGACCTA      360
                    ************************************************************

XM_025282898.3      ACCATCAAGCTGCCTGATGGATACGAATTCAAGTT   395
H                   ACCATCAAGCTGCCTGATGGATACGAATTCAAGTT   395
RD                  ACCATCAAGCTGCCTGATGGATACGAATTCAAGTT   395
                    ***********************************
```

Figure S2. Analysis of nitrogenous bases matching for DNA in healthy and reproductive disorders affected buffaloes using GenBank gb|XM_025282898.3| and *LGALS* marker (395-bp) sequences.

```
NM_001290898.1      CCATGGCAACCGTACCTGAACCCATCAATGAAATGATGGCCTACTACAGTGACGAGAATG      60
H                   CCATGGCAACCGTACCTGAACCCATCAATGAAATGATGGCCTACTACAGTGATGAGAATG      60
RD                  CCATGGCAACCGTACCTGAACCCATCAATGAAATGATGGCCTACTACAGTGACGAGAATG      60
                    **************************************************** *******

NM_001290898.1      AGCTGTTATTTGAGGCTGATGGCCCCAAACAGATGAAGAGCTGCATCCAACACCTGGACC      120
H                   AGCTGTTATTTGAGGCTGATGGCCCCAAACAGATGAAGAGCTGCATCCAACACCTGGACC      120
RD                  AGCTGTTATTTGAGGCTGATGGCCCCAAACAGATGAAGAGCTGCATCCAACACCTGGACC      120
                    ************************************************************

NM_001290898.1      TCGGTTCCATGGGAGATGGAAACATCCAGCTGCAGATTTCTCACCAGCTCTACAACAAAA      180
H                   TCGGCTCCATGGGAGATGGAAACATCCAGCTGCAGATTTCTCACCAGCTCTACAACAAAA      180
RD                  TCGGTTCCATGGGAGATGGAAACATCCAGCTGCAGATTTCTCACCAGCTCTACAACAAAA      180
                    **** *******************************************************

NM_001290898.1      GCTTCAGGCAGGTGGTGTCGGTCATTGTGGCCATGGAGAAGCTGAGGAACAGTGCCTACG      240
H                   GCTTCAGGCAGGTGGTGTCGGTCATTGTGGCCATGGAGAAGCTGAGGAACAGTGCCTACG      240
RD                  GCTTCAGGCAGGTGGTGTCGGTCATCGTGGCCATGGAGAAGCTGAGGAACAGTGCCTACG      240
                    ************************* **********************************

NM_001290898.1      CACATGTCTTCCATGATGATGACCTGAGGAGCATCCTTTCATTCATCTTTGAAGAAGAGC      300
H                   CACATGTCTTCCATGATGATGACCTGAGGAGCATCCTTTCATTCATCTTTGAAGAAGAGC      300
RD                  CACATGTCTTCCATGATGATGACCTGAGGAGCATCCTTTCATTCATCTTTGAAGAAGAGC      300
                    ************************************************************

NM_001290898.1      CTGTCATCTTCGAAACGTCCTCCGACGAGTTTCTGTGTGACGCACCCGTGCAGTCAGT        358
H                   CTGTCATCTTTGAAACGTCCTCCGACGAGTTTCTGTGTGACGCACCCGTGCAGTCAGT        358
RD                  CTGTCATCTTCGAAACGTCCTCCGACGAGTTTCTGTGTGACGCACCCGTGCAGTCAGT        358
                    ********** ***********************************************
```

Figure S3. Analysis of nitrogenous bases matching for DNA in healthy and reproductive disorders affected buffaloes using GenBank gb|NM_001290898.1| and *IL1B* marker (358-bp) sequences.

```
HQ889748.1      CTTGAATCCTCTCGCTGCAACATGAAGGTCTCCGCTGCCCTCCTGTGCCTGCTACTCACA 60
H               CTTGAATCCTCTCGCTGCAACATGAAGGTCTCCGCTGCCCTCCTGTGCCTGCTACTCACA 60
RD              CTTGAATCCTCTCGCTGCAACATGAAGGTCTCCGCTGCCCTCCTGTGCCTGCTACTCACA 60
                ************************************************************

HQ889748.1      GTAGCTGCCTTCAGCACCGAGGTGCTCGCTCAGCCAGATGCAATTAACTCCCAAGTCGCC 120
H               GTAGCTGCCTTCAGCACCGAGGTGCTCGCTCAGCCAGATGCAATTAACTCCCAAGTCGCC 120
RD              GTAGCTGCCTTCAGCACCGAGGTGCTCGCTCAGCCAGATGCAATTAACTCCCAAGTCGCC 120
                ************************************************************

HQ889748.1      TGCTGCTATACATTCAACAGTAAGAAGATCTCCATGCAGAGGCTGATGAACTACAGAAGA 180
H               TGCTGCTATACATTCAACAGTAAGAAGATCTCCATGCAGAGGCTGATGAACTACAGAAGA 180
RD              TGCTGCTATACATTCAACAGTAAGAAGATCTCCATGCAGAGGCTGATGAACTACAGAAGA 180
                ************************************************************

HQ889748.1      GTCACCAGCAGCAAGTGTCCCAAAGAGGCTGTGATTTTCAAGACCGTCCTGGGCAAGGAG 240
H               GTCACCAGCAGCAAGTGTCCCAAAGAGGCTGTGATTTTCAAGACCGTCCTGGGCAAGGAG 240
R               GTCACCAGCAGCAAGTGTCCTAAAGAGGCTGTGATTTTCAAGACCGTCCTGGGCAAGGAG 240
                ******************** ***************************************

HQ889748.1      TTATGTGCAGACCCCAAGCAGAAATGGGTCCAGGACTCCATAAACTATCTCAACAAGAAA 300
H               TTATGTGCAGACCCCAAGCAGAAATGGGTCCAGGACTCCATAAACTATCTCAACAAGAAA 300
RD              TTATGTGCAGACCCCAAGCAGAAATGGGTCCAGGACTCCATAAACTATCTCAACAAGAAA 300
                ************************************************************

HQ889748.1      AACCAAACTCCGAAGCCTTGAGCACTCACTCCACAACCCAAGAATCT    347
H               AACCAAACTCCGAAGCCTTGAGCACTCACTCCACAACCCAAGAATCT    347
RD              AACCAAACTCCGAAGCCTTGAGCACTCACTCCACAACCCAAGAATCT    347
                ***********************************************
```

Figure S4. Analysis of nitrogenous bases matching for DNA in healthy and reproductive disorders affected buffaloes using GenBank gb|HQ889748.1| and *CCL2* marker (347-bp) sequences.

```
XM_006056212.4      CAGCCGGAGCTGCAGAGGATCAGCACGCGGATCGCCCAGCCTCTGCCCACAGCTACCATG      60
H                   CAGCCGGAGCTGCAGAGGATCAGCACGCGGATCGCCCAGCCTCTGCCCACAGCTACCATG      60
RD                  CAGCCGGAGCTGCAGAGGATCAGCACGCGGATCGCCCAGCCTCTGCCCACAGCTACCATG      60
                    ************************************************************

XM_006056212.4      AAGGTCTCCGCCGCTGCCCTCGCTGTCCTCCTGACGGTGGCCGCCCTCTGCGCTCCTGCG      120
H                   AAGGTCTCCGCCGCTGCCCTCGCTGTCCTCCTGACGGTGGCCGCCCTCTGCGCTCCTGCG      120
RD                  AAGGTCTCCGCCGCTGCCCTCGCTGTCCTCCTGACCGTGGCCGCCCTCTGCGCTCCTGCG      120
                    *********************************** ************************

XM_006056212.4      TCTGCCTCCCCATATGCCTCAGACACCACGCCCTGCTGCTTTGCCTACATCTCCCGCCCG      180
H                   TCTGCCTCCCCATATGCCTCAGACACCACGCCCTGCTGCTTTGCCTACATCTCCCGCCCG      180
RD                  TCTGCCTCCCCATATGCCTCGGACACCACGCCCTGCTGCTTTGCCTACATCTCCCGCCCG      180
                    ******************** ***************************************

XM_006056212.4      CTGCCCCGCACCCACGTCCAGGAGTATTTCTACACCAGCAGCAAGTGCTCCATGGCAGCA      240
H                   CTGCCCCGCACCCACGTCCAGGAGTATTTCTACACCAGCAGCAAGTGCTCCATGGCAGCA      240
RD                  CTGCCCCGCACCCACGTCCAGGAGTATTTCTACACCAGCAGCAAGTGCTCCATGGCAGCA      240
                    ************************************************************

XM_006056212.4      GTTGTCTTTATCACCAGGAAGAACCGCCAGGTGTGCGCCAACCCAGAGAAGAAGTGGGTG      300
H                   GTTGTCTTTATCACCAGGAAGAACCGCCAGGTGTGCGCCAACCCAGAGAAGAAGTGGGTG      300
RD                  GTTGTCTTTATCACCAGGAAGAACCGCCAGGTGTGCGCCAACCCAGAGAAGAAGTGGGTG      300
                    ************************************************************

XM_006056212.4      CGAGAGTACATCAACGCTTTGGAGTTGAGCTAGGGTGGAGGACGCCTTGAACCTGAACTT      360
H                   CGAGAGTACATCAACGCTTTGGAGTTGAGCTAGGGTGGAGGACGCCTTGAACCTGAACTT      360
RD                  CGAGAGTACATCAACGCTTTGGAGTTGAGCTAGGGTGGAGGACGCCTTGAACCTGAACTT      360
                    ************************************************************

XM_006056212.4      GCGCCAACTCTGCTTCTCGCTCTTGTCCTAAGCAGCTTG        399
H                   GCGCCAACTTTGCTTCTCGCTCTTGTCCTAAGCAGCTTG        399
RD                  GCGCCAACTCTGCTTCTCGCTCTTGTCCTAAGCAGCTTG        399
                    ********* *****************************
```

Figure S5. Analysis of nitrogenous bases matching for DNA in healthy and reproductive disorders affected buffaloes using GenBank gb|XM_006056212.4| and *RANTES* marker (399-bp) sequences.

```
XR_006551148.1      AGAGCACAGACACAGAGCGGGCGCCTGGCAACGACACCTTCTACTCACCGGGCTCCAGCC      60
H                   AGAGCACAGACACAGAGCGGGCGCCTGGCAACGACACCTTCTACTCACCGGGCTCCAGCC      60
RD                  AGAGCACAGACACAGAGCGGGCGCCTGGCAACGACACCTTCTACTCACCGGGCTCCAGCC      60
                    ************************************************************

XR_006551148.1      TGGATGTCACCTTCCGCTCCGACTACTCCAACGAGAAGCAGTTCACAGGCTTCGAGGCCT      120
H                   TGGATGTCACCTTCCGCTCCGACTACTCCAACGAGAAGCAGTTCACAGGCTTCGAGGCCT      120
RD                  TGGATGTCACCTTCCGCTCCGACTACTCCAACGAGAAGCAGTTCACAGGCTTCGAGGCCT      120
                    ************************************************************

XR_006551148.1      TCTACTCTGCAGAGGACATTGACGAGTGCCAGGTGCCCCCAGGAGAGGCCCCCACCTGCG      180
H                   TCTACTCTGCAGAGGACATTGACGAGTGCCAGGTGCCCCCAGGAGAGGCCCCCACCTGCG      180
RD                  TCTACTCTGCAGAGGACATTGACGAGTGCCAGGTGCCCCCAGGAGAGGCCCCCACCTGCG      180
                    ************************************************************

XR_006551148.1      ACCACCACTGCCACAACCACCTGGGCGGCTTCTACTGCTCCTGCCGTGTGGGCTATGTTC      240
H                   ACCACCACTGCCACAACCACCTGGGCGGCTTCTACTGCTCCTGCCGTGTGGGCTATGTTC      240
RD                  ACCACCACTGCCACAACCACCTGGGCGGCTTCTACTGCTCCTGCCGTGTGGGCTATGTTC      240
                    ************************************************************

XR_006551148.1      TCCACAGGAACAAGCGCACCTGCTCAGATTCAAACAGACAAGGCGGAATACGGCCCGTTC      300
H                   TCCACAGGAACAAGCGCACCTGCTCAGATTCAAACAGACAAGGCGGAATACGGCCCGTTC      300
RD                  TCCACAGGAACAAGCGCACCTGCTCAGATTCAAACAGACAAGGCGGAATACGGCCCGTTC      300
                    ************************************************************

XR_006551148.1      TGTGGGAAGACATTGCCCAGCAGGATTGAAACCAAGAGCAATACCGTGACCATCACCTTT      360
H                   TGTGGGAAGACATTGCCCAGCAGGATTGAAACCAAGAGCAATACCGTGACCATCACCTTT      360
RD                  TGTGGGAAGACATTGCCCAGCAGGATTGAAACCAAGAGCAATACCGTGACCATCACCTTT      360
                    ************************************************************

XR_006551148.1      ACCACCGATCAGTCGGGGGACCACATGGGCTGGAAGGTCCGATACAACAGCACAGACTTG      420
H                   ACCACCGATCAGTCGGGGGACCACATGGGCTGGAAGGTCCGATACAACAGCACAGACTTG      420
RD                  ACCACCGATCAGTCGGGGGACCACATGGGCTGGAAGGTCCGATACAACAGCACAGACTTG      420
                    ************************************************************

XR_006551148.1      CCTGCTGTAGACCACAGCCAAACCCATACGTCTCATTTCCTTGCTCAGCTGAGCCTTGCC      480
H                   CCTGCTGTAGACCACAGCCAAACCCATACGTCTCATTTCCTTGCACAGCTGAGCCTTGCC      480
RD                  CCTGCTGTAGACCACAGCCAAACCCATACGTCTCATTTCCTTGCTCAGCTGAGCCTTGCC      480
                    ******************************************* ****************

XR_006551148.1      CTGATCCATTGGCGCCACCTAATGGCCGCATCT     513
H                   CTGATCCATTGGCGCCACCTAATGGCCGCATCT     513
RD                  CTGATCCATTGGCGCCACCTAATGGCCGCATCT     513
                    *********************************
```

Figure S6. Analysis of nitrogenous bases matching for DNA in healthy and reproductive disorders affected buffaloes using GenBank gb|XR_006551148.1| and *MASP2* marker (513-bp) sequences.

```
XM_006078587.2      CGCTGGCTGGAGAGTAATGTTACAGAGCGGAGAGAGTGAGGAGGCTGCGTCTGGCTCCCG      60
H                   CGCTGGCTGGAGAGTAATGTTACAGAGCGGAGAGAGTGAGGAGGCTGCGTCTGGCTCCCG      60
RD                  CGCTGGCTGGAGAGTAATGTTACAGAGCGGAGAGAGTGAGGAGGCTGCGTCTGGCTCCCG      60
                    ************************************************************

XM_006078587.2      CTCTCACAGCCATTGCAGTACATTGAGCTCCATAGAGACAGCGCCGGGGCAAGTGAGAGC      120
H                   CTCTCACAGCCATTGCAGTACATTGAGCTCCATAGAGACAGCGCCGGGGCAAGTGAGAGC      120
RD                  CTCTCACAGCCATTGCAGTACATTGAGCTCCATAGAGACAGCGCCGGGGCAAGTGAGAGC      120
                    ************************************************************

XM_006078587.2      CGGACGGGCACTGGGCGACTCTGTGCCTCGCGGAGGAAAAATAATTAAACATGGGCAAAG      180
H                   CGGACGGGCACTGGGCGACTCTGTGCCTCGCGGAGGAAAAATAATTAAACATGGGCAAAG      180
RD                  CGGACGGGCACTGGGCGACTCTGTGCCTCGCGGAGGAAAAATAATTAAACATGGGCAAAG      180
                    ************************************************************

XM_006078587.2      GAGATCCTAAGAAGCCGAGAGGCAAAATGTCATCATATGCATTCTTCGTGCAAACTTGCC      240
H                   GAGATCCTAAGAAGCCGAGAGGCAAAATGTCATCATATGCATTCTTTGTGCAAACTTGCC      240
RD                  GAGATCCTAAGAAGCCGAGAGGCAAAATGTCATCATATGCATTCTTCGTGCAAACTTGCC      240
                    ********************************************** *************

XM_006078587.2      GGGAGGAGCACAAGAAGAAGCACCCGGATGCTTCCGTCAACTTCTCAGAGTTTTCTAAGA      300
H                   GGGAGGAGCACAAGAAGAAGCACCCGGATGCTTCCGTCAACTTCTCAGAGTTTTCTAAGA      300
RD                  GGGAGGAGCACAAGAAGAAGCACCCGGATGCTTCTGTCAACTTCTCAGAGTTTTCTAAGA      300
                    ********************************** *************************

XM_006078587.2      AGTGCTCAGAGAGGTGGAAGACCATGTCTGCTAAAGAGAAAGGAAAATTTGAAGACATGG      360
H                   AGTGCTCAGAGAGGTGGAAGACCATGTCTGCTAAAGAGAAAGGAAAATTTGAAGACATGG      360
RD                  AGTGCTCAGAGAGGTGGAAGACCATGTCTGCTAAAGAGAAAGGAAAATTTGAAGACATGG      360
                    ************************************************************

XM_006078587.2      CAAAGGCGGACAAGGCCCGTTATGAAAGAGAAATGAAAACTTATATTCCTCCTAAAGGGG      420
H                   CAAAGGCGGACAAGGCCCGTTATGAAAGAGAAATGAAAACTTATATCCCTCCTAAAGGGG      420
RD                  CAAAGGCGGACAAGGCCCGTTATGAAAGAGAAATGAAAACTTATATTCCTCCTAAAGGGG      420
                    ********************************************** *************

XM_006078587.2      AAACAAAAAAGAAGTTCAAGGATCCCAATGCACCCAAGAGGCCTCCTTCGGCCTTTTTCT      480
H                   AAACAAAAAAGAAGTTCAAGGATCCCAATGCACCCAAGAGGCCTCCTTCGGCCTTTTTCT      480
RD                  AAACAAAAAAGAAGTTCAAGGATCCCAATGCACCCAAGAGGCCTCCTTCGGCCTTTTTCT      480
                    ************************************************************

XM_006078587.2      TGTTTTGTTCTGAGTATCGTCCAAAAATCAAAGGCGAACATCCTGGCCTGTCTATTGGTG      540
H                   TGTTTTGTTCTGAGTATCGTCCAAAAATCAAAGGCGAACATCCTGGCCTGTCTATTGGTG      540
RD                  TGTTTTGTTCTGAGTATCGTCCAAAAATCAAAGGCGAACATCCTGGCCTGTCTATTGGTG      540
                    ************************************************************
```

Figure S7. Analysis of nitrogenous bases matching for DNA in healthy and reproductive disorders affected buffaloes using GenBank gb|XM_006078587.2| and *HMBG1* marker (540-bp) sequences.

```
EF535848.1      TCTGGACATTTGACTTCTCAGTCAACATGAAGGCTCTCATTATTCTGGGGTTTCTCTTCC 60
H               TCTGGACATTTGACTTCTCAGTCAACATGAAGACTCTCATTATTCTGGGGTTTCTCTTCC 60
RD              TCTGGACATTTGACTTCTCAGTCAACATGAAGGCTCTCATTATTCTGGGGTTTCTCTTCC 60
                ******************************** ***************************

EF535848.1      TTTCTGTTGCTGTCCAAGGCAAGGTCTTTGAGAGATGTGAGCTTGCCAGAACGCTGAAGA 120
H               TTTCTGTTGCTGTCCAAGGCAAGGTCTTTGAGAGATGTGAGCTTGCCAGAACGCTGAAGA 120
RD              TTTCTGTTGCTGTCCAAGGCAAGGTCTTTGAGAGATGTGAGCTTGCCAGAACGCTGAAGA 120
                ************************************************************

EF535848.1      AACTTGGACTGGACGGCTATAAGGGAGTCAGCCTGGCGAACTGGTTGTGTTTGACCAAAT 180
H               AACTTGGACTGGACGGCTATAAGGGAGTCAGCCTGGCGAACTGGTTGTGTTTGACCAAAT 180
RD              AACTTGGACTGGACGGCTATAAGGGAGTCAGCCTGGCGAACTGGTTGTGTTTGACCAAAT 180
                ************************************************************

EF535848.1      GGGAAAGCAGTTATAACACAAAAGCTACAAACTACAATCCTAGCAGTGAAAGCACTGATT 240
H               GGGAAAGCAGTTATAACACAAAAGCTACAAACTACAATCCTAGCAGTGAAAGCACTGATT 240
RD              GGGAAAGCAGTTATAACACAAAAGCTACAAACTACAATCCTGGCAGTGAAAGCACTGATT 240
                ***************************************** ******************

EF535848.1      ATGGGATATTTCAGATCAACAGCAAATGGTGGTGTAATGATGGCAAAACCCCTAACGCAG 300
H               ATGGGATATTTCAGATCAACAGCAAATGGTGGTGTAATGATGGCAAAACCCCTAACGCAG 300
RD              ATGGGATATTTCAAATCAACAGCAAATGGTGGTGTAATGATGGCAAAACCCCTAACGCAG 300
                ************ ***********************************************

EF535848.1      TTGACGGCTGTCATGTATCCTGCAGCGAATTAATGGAAAATGACATCGCTAAAGCTGTAG 360
H               TTGACGGCTGTCATGTATCCTGCAGCGAATTAATGGAAAATGACATCGCTAAAGCTGTAG 360
RD              TTGACGGCTGTCATGTATCCTGCAGCGAATTAATGGAAAATGACATCGCTAAAGCTGTAG 360
                ************************************************************

EF535848.1      CCTGTGCAAAGCAGATCGTCAGTGAGCAAGGCATTACAGCATGGGTGGCATGGAAAAGTC 420
H               CCTGTGCAAAGCAGATCGTCAGTGAGCAAGGCATTACAGCATGGGTGGCATGGAAAAGTC 420
RD              CCTGTGCAAAGCAGATCGTCAGTGAGCAAGGCATTACAGCATGGGTGGCATGGAAAAGTC 420
                ************************************************************

EF535848.1      ATTGTCGAGACCATGACGTCAGCAGTTACATTGAG          455
H               ATTGTCGAGACCATGACGTCAGCAGTTACATTGAG          455
RD              ATTGTCGAGACCATGACGTCAGCAGTTACATTGAG          455
                ***********************************
```

Figure S8. Analysis of nitrogenous bases matching for DNA in healthy and reproductive disorders affected buffaloes using GenBank gb|EF535848.1| and *S-LZ* marker (455-bp) sequences.
